# Exploration of Protease Resources in the Gut of Omnivorous *Gryllotalpa orientalis* (Orthoptera: Gryllotalpidae)

**DOI:** 10.3390/biology13090650

**Published:** 2024-08-23

**Authors:** Xiang Zheng, Fangtong Wu, Lu Zhao, He Zhou, Zhijun Zhou, Zhenhua Jia, Fuming Shi

**Affiliations:** 1 Laboratory of Enzyme Preparation, Hebei Research Institute of Microbiology Co., Ltd., Baoding 071051, China; 569186912@163.com (X.Z.); yayawu710@hotmail.com (F.W.); 15832297069@139.com (L.Z.); zhouhebio@163.com (H.Z.); zhenhuaj@hotmail.com (Z.J.); 2 College of Life Science, Institute of Life Science and Green Development, Hebei University, Baoding 071002, China; zhijunzhou@hbu.edu.cn; 3 Institute of Biology, Hebei Academy of Sciences, Shijiazhuang 050052, China

**Keywords:** insect, gut protease, culture independent, gut bacteria, recombinant

## Abstract

**Simple Summary:**

The digestive enzymes present in the insect gut play a crucial role in digesting food and extracting essential nutrients. The diverse range of digestive enzymes found in the insect gut serves as a valuable source of functional proteins. With a significant population of herbivorous insects, there has been extensive research on cellulase in the insect gut. This study utilized both culture-independent and culture-dependent methods to investigate the protease resources responsible for digesting animal and plant proteins, laying a solid foundation for future research and utilization of proteases derived from insect sources.

**Abstract:**

An insect’s gut microbiome is an essential “organ” in their life cycle, playing a crucial role by aiding food digestion and nutrient absorption. This study employed both culture-independent and culture-dependent methods to explore the protease resources present in the gut of the omnivorous insect *Gryllotalpa orientalis*. The findings revealed that the gut extract of *G. orientalis* contained a diverse array of proteases, including cysteine proteases, pepsin, serine proteases, and trypsin, as well as some unidentified proteases. Furthermore, the protease gene *htpX*, derived from gut bacterium *Priestia megaterium* DX-3, has been cloned and recombinantly expressed. The recombinant DX-3-*htpX* protease exhibited a 61.9-fold increase in fermentation level compared to the DX-3 protease. This protease was characterized as a neutral, heat-resistant metalloprotease with an M48 peptidase domain, and it was observed that the binding of Ca^2+^ to the recombinant protease resulted in the formation of the largest active pocket. This study provides technical support for further development and utilization of functional protein resources in insect gut.

## 1. Introduction

The gut of insects is rich in microbial resources, and this diverse gut environment is home to highly diverse species and biological functions, making it a valuable resource for discovering new genes and active substances [1]. The gut microbiota plays a crucial role in helping insect hosts digest food and absorb nutrients [2], even improving the digestive ability of insects to quickly adapt to environmental changes in the face of food scarcity [3]. Nutrient supply is attained through the secretion of various necessary hydrolytic enzymes [4], such as lignocellulase [5], xylanase, protease [6], amylase, lipase [7], and pectinase, et al., for nutrient decomposition, digestion, and absorption.

Various enzyme-producing microorganisms have been screened and identified based on studying the gut of insects [8]. Due to the high proportion of insect species that feed on plant materials, screening enzyme-producing microorganisms in the gut mainly requires the study of lignocellulose-degrading enzymes [9]. Relevant research has been carried out on various insects, such as Coleoptera, Lepidoptera, Hemiptera, and Isopteran. However, the gut of insects also possesses various proteases with superior catalytic performance and stable chemical properties, such as trypsin, chymotrypsin, elastase, cathepsin, aminopeptidase, carboxypeptidase, and serine protease [10], which play an essential role in the degradation and immunity of animal and plant proteins. Although microbial culture technology is the most direct means of screening functional microorganisms from nature, exploring novel microbial enzyme resources by using non-cultivation techniques is becoming increasingly popular due to the currently non-cultivable characteristics of most microorganisms. For a considerable number of unknown digestive enzymes in the insect gut, the gene sequence, classification, molecular weight, and functional information of the enzymes can be clarified through enzyme inhibitors [11], zymography [12], chromatography [13], mass spectrometry [11], proteomics [14], and metagenomics [15]. Microbial proteases can be used for the clean production of leather, for enhancing food flavors, as washing aids, and in silk degumming processes [16]. Therefore, proteases derived from insects’ guts have excellent potential for application.

This study utilized culture-independent and recombinant protein expression technology to explore protease resources from the gut of the omnivorous *Gryllotalpa orientalis* in Gryllotalpidae. This study provides a foundation for the application and development of insect gut resources for addressing issues in agriculture, industry, and environmental protection [17].

## 2. Materials and Methods

### 2.1. Analysis of Proteases Derived from Gut Extract

The *G. orientalis* were dissected under sterile conditions to obtain a solution of gut contents. After centrifugation at 6000 r/min at 4 °C for 30 min, the supernatant was collected for subsequent analysis of the gut proteins. The protein concentration of the gut extract was determined using a BCA protein concentration determination method. The protease activity of the gut extract was determined using the Folin–Ciocalteu method [18].

Zymography analysis [16,19]: Zymogram gel electrophoresis was conducted by applying gelatin with a final concentration of 0.1% to the separation gel, though β-mercaptoethanol was not used. We mixed each protein sample with denatured non-reducing loading buffer in a 4:1 ratio and left the mixture at room temperature for 5–10 min without boiling. After sample loading, electrophoresis was carried out at 150 V under ice-bath conditions. Then, the protein gel was first slowly shaken in 50 mL distilled water for 5 min and transferred to a pH 7.6 renaturation buffer containing 2.5% Triton X-100, 50 mM Tris-HCl, and 5 mM CaCl_2_, before being slowly shaken for 30 min. Then, it was transferred to a pH 7.5 incubation buffer containing 50 mM Tris-HCl, 150 mM NaCl, 10 mM CaCl_2_, and 0.02% NaN_3_ overnight and slowly shaken at 37 °C. Afterwards, protein gel staining and decolorization analysis were performed.

Protease inhibitor analysis [11,20]: We prepared 100 mM PMSF (phenylmethanesulfonyl fluoride, a serine protease inhibitor) solution, 30 mM TPCK (*N*-p-Tosyl-L-phenylalanine chloromethyl ketone, a chymotrypsin inhibitor) solution, 10 mM IAA (indoleacetic acid, a cysteine protease inhibitor) solution, 1 mM E-64 (*N*-(trans-epoxysuccinyl)-L-leucine 4-guanidinobutylamide, a cysteine protease inhibitor) solution, 0.1 mM EGTA (ethylene glycol-bis (2-aminoethylether)-*N*,*N*,*N*′,*N*′-tetraacetic acid, a metalloprotease inhibitor) solution, 1 mM pepsin inhibitor (an aspartate protease inhibitor) solution, and 0.2 mg/mL trypsin protease inhibitor solution. Eight portions of 100 μL prepared insect gut protein samples were taken and mixed with the protease inhibitor at the specified concentration in a ratio of 1:100. Additionally, 1 μL of sterile distilled water was added to one portion as a control. Then, zymography gel electrophoresis was conducted to observe the inhibition of gut protease activity and preliminarily infer the protease information.

LC-MS/MS (Liquid chromatography–mass spectrometry) analysis [21,22]: We performed gel-cutting mass spectrometry on the target protease bands in protein gels. Then, enzymatic hydrolysis, liquid chromatography, and mass spectrometry analysis of the target protein gels were performed at Beijing Protein Innovation Co., Ltd. (Beijing, China). The steps were as follows: We washed the protein gel twice with ddH_2_O and added 1 mL of digestion solution containing 50% acetonitrile and 25 mM NH_4_HCO_3_, cleaning twice more. We added acetonitrile to dehydrate and whiten the protein gel. The gel was incubated in the presence of 10 mM DTT at 56 °C for 1 h, placed in a dark room with 55 mM IAM for 45 min, washed twice with 25 mM NH_4_HCO_3_, and then rinsed twice with decolorization solution. We added acetonitrile to completely whiten the protein gel and added an enzyme storage solution with NH_4_HCO_3_ to a water bath at 37 °C, where it was kept overnight. Finally, 0.1% FA at the final concentration was added to terminate the reaction. The peptide segment was separated using an ultra-high-performance liquid chromatography system and subjected to LC-MS/MS analysis. The mass spectrometry database we used was Uniprot 20160315 (https://www.uniprot.org/, accessed on 9 February 2023), and the data were retrieved and analyzed using Mascot Server 2.3.0 (https://www.matrixscience.com, accessed on 10 February 2023). 

### 2.2. Analysis of Proteases Derived from Gut Bacteria

Fermentation was conducted on *Priestia megaterium* (DX-3), a strain derived from the insect gut with protease activity, focusing on analyzing the properties of the proteases in a fermentation medium containing 3% maltose and 3% skimmed milk powder. We collected fermentation supernatants for 20, 28, 36, and 44 h and analyzed the optimal temperature and pH, as well as the tolerance of the DX-3 protease to the temperature and pH. Meanwhile, the fermentation supernatant was collected for SDS-PAGE electrophoresis analysis.

The protease gene *htpX* was obtained from the whole genome sequencing of strain DX-3. Primers containing recognition sites for the restriction endonucleases *BamH* I and *Sma* I were designed based on the gene sequence of *htpX*, including P1:5′-CGGATC CTG CTGCTAAAACATTCACTGTT-3′ and P2:5′-TCCCCGGGTTTATAGGAATGCAAGCGC-3′. The genomic DNA of strain DX-3 was as a template, and the protease gene *htpX* was amplified by PCR. After recovering the PCR amplification product, the vector pHT43 was treated with T4 ligase overnight, digested with *BamH* I and *Sma* I at 16 °C, and transformed into *Escherichia coli* DH5α, before being validated through bacterial PCR and sequencing. The recombinant plasmid pHT43-*htpX* was further transformed into *E. coli* BL21 (DE3) to improve transformation efficiency. After successful transformation, it was electro-transformed into *Bacillus subtilis* WB800N and coated on LB plates containing Cm resistance to screen positive transformants. We inoculated the engineering strain WB800N/pHT43-*htpX* in LB medium with Cm at a final concentration of 10 μg/mL cultured it to OD600 ≈ 0.6–0.8 and added IPTG with a final concentration of 1 mM to induce recombinant protein expression. In contrast, no IPTG was added to the control. After centrifugation, the fermentation supernatant was obtained and subjected to SDS-PAGE electrophoresis analysis, and enzymatic properties analysis of the recombinant protease DX-3-*htpX* was also carried out.

Tertiary structure prediction and D3 pocket analysis of the DX-3-*htpX* protease: The conserved domain was analyzed using the InterPro server (http://www.ebi.ac.uk/interpro/, accessed on 10 November 2023) [23]. The tertiary structure was predicted by AlphaFold3 [24]. The D3 pocket and its binding to metal ions were analyzed using CASTpFold (http://sts.bioe.uic.edu/castp/index.html, accessed on 20 May 2024) [25]. The selection of binding pockets was based on prominent concave protein regions frequently associated with binding events, utilizing the alpha shape and the pocket algorithm developed in computational geometry. Subsequently, PyMOL was used to display the tertiary structure of the DX-3-*htpX* protease. 

### 2.3. Statistical Analysis

All experiments were conducted in triplicate, and the results are reported as the mean value ± SD. Line graphs were generated using Origin 2021.

## 3. Results

### 3.1. Exploration of Protease Resources from the Gut Extract

The protein content and protease activity of the gut protein samples were determined to be 39.43 ± 0.84 mg/g and 608.19 ± 11.11 U/g. These results indicated that the protease resources in the gut extract of *G. orientalis* could be effectively utilized for further exploitation and research purposes.

**Figure 1:**
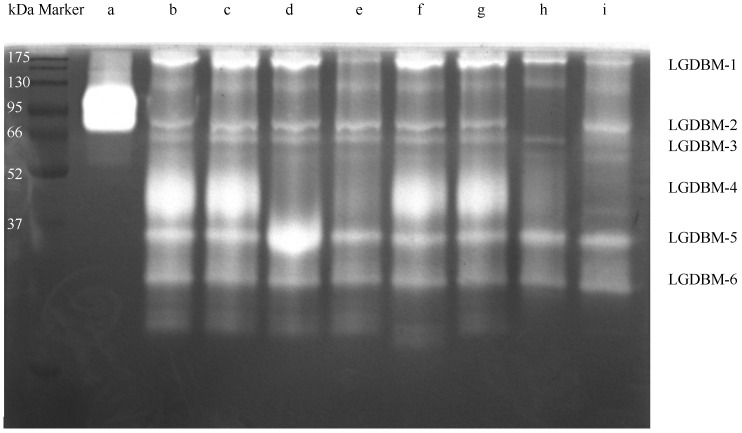
The zymography analysis of the gut protein sample of *G. orientalis*. (a) Collagenase. (b) Gut protein samples. (c) Gut extract with TPCK. (d) Gut extract with E-64. (e) Gut extract with Pepsin inhibitor. (f) Gut extract with EGTA. (g) Gut extract with IAA. (h) Gut extract with PMSF. (i) Gut extract with Trypsin inhibitor.

After low-temperature electrophoresis and substrate co-bath treatment, the protease in the gut extract of *G. orientalis* hydrolyzed the gelatin substrate in the gel. The light-colored area of the gel indicated that a protein hydrolysis reaction had occurred (Figure 1 and Figure S1). The most highly active protein had a molecular weight of approximately 45 kDa, and proteins with relatively high protease activity had molecular weight values of 28, 35, 60, 66, and 200 kDa. The zymogram gel electrophoresis results derived from mixing different protease inhibitors with gut extract samples showed that TPCK, EGTA, and IAA protease inhibitors had no inhibitory effect on the gut protease of *G. orientalis*. E-64, PMSF, pepsin inhibitors, and trypsin inhibitors had varying degrees of inhibitory effects on the gut proteases. E-64 exerted an inhibitory effect on the LGDBM-4 protease but had an activation effect on the LGDBM-5 protease. PMSF exerted and inhibitory effect on the LGDBM-2 and LGDBM-4 proteases and a slight inhibitory effect on the LGDBM-1 protease. Pepsin inhibitors had an inhibitory effect on the LGDBM-4 protease and a slight inhibitory effect on the LGDBM-1 protease. Trypsin inhibitors exerted an inhibitory effect on the LGDBM-4 protease, a slight inhibitory effect on the LGDBM-1 and LGDBM-3 proteases, and an activation effect on LGDBM-2 protease.

Overall, the LGDBM-1 protease was affected by pepsin inhibitors, PMSF, and trypsin inhibitors, suggesting that its components contain pepsin, serine protease, and trypsin. The LGDBM-2 protease was affected by PMSF, suggesting that its components contain serine protease. The LGDBM-3 protease was affected by trypsin inhibitors, suggesting that its components contain trypsin. The LGDBM-4 protease was affected by E-64, pepsin inhibitors, PMSF, and trypsin inhibitors, suggesting that its components contain cysteine protease, pepsin, serine protease, and trypsin. Various protease inhibitors did not inhibit the LGDBM-5 and LGDBM-6 proteases mentioned above, and E-64 activated the LGDBM-5 protease; their composition needs further analysis and identification (Table 1). 

Mass spectrometry analysis of protein bands with protease activity in the zymogram gel showed that LGDBM-1, LGDBM-2, LGDBM-3, LGDBM-4, LGDBM-5, and LGDBM-6 were identified as having 101, 29, 60, 59, 52, and 58 proteins, and they contained 19, 8, 12, 18, 14, and 19 protein modification sites, respectively. Based on the Uniprot database, protein information related to the protease activity exhibited by each protein is shown in Table 2. Except for the LGDBM-2 protease, all other protein samples showed protease-related information upon mass spectrometry identification. The glutathione S-transferase (Uniprot ID: P08515) acted on glutathione and dermcidin (Uniprot ID: P81605), mainly hydrolyzed arginine, and was detected in all protein samples. Meanwhile, specific types of proteases were also found, such as proteasome subunit alpha type-6 (Uniprot ID: P28072) in LGDBM-1, which is a component of the core proteasome complex and participates in the proteolytic degradation of most intracellular proteins. The ATP-dependent zinc metalloprotease (Uniprot ID: Q8W585) in LGDBM-3 is a part of the ATP-dependent zinc metalloproteinase complex. Gamma-glutamyl cyclotransferase (Uniprot ID: Q32LE4) in LGDBM-3 catalyzed the formation of 5-oxo proline from gamma-glutamyl dipeptides and may play an essential role in glutathione homeostasis. Cathepsin D (Uniprot ID: Q4LAL9) in LGDBM-4 belongs to the peptidase family A1 and functions similarly to pepsin A [26]. The proteases detected in LGDBM-5 and LGDBM-6 can also be found in the other protein samples, and no unique protease information was detected. 

### 3.2. Exploration of Protease from Gut Bacteria

**Figure 2:**
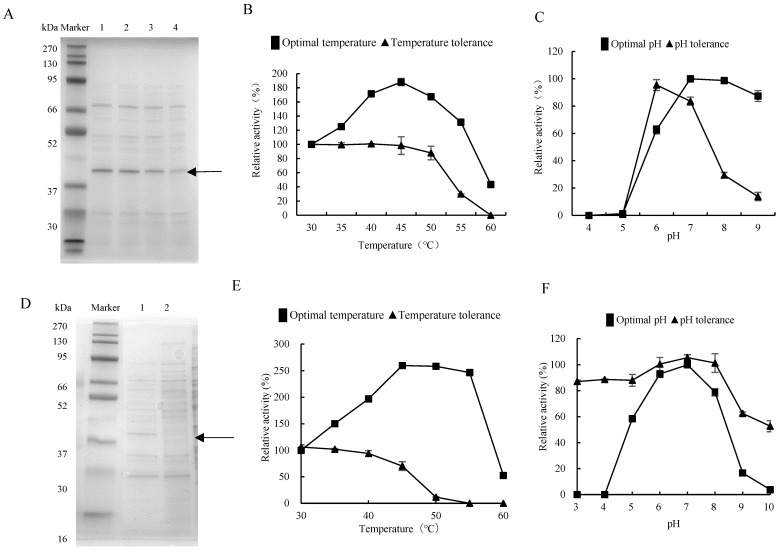
Enzymatic properties of proteases from gut bacteria. (**A**) SDS-PAGE analysis of the DX-3 proteases with different fermentation durations. Lanes 1–4 were fermented for 44, 36, 28, and 20 h, respectively. (**B**) Optimal temperature and temperature tolerance of the DX-3 protease. (**C**) Optimal pH and pH tolerance of the DX-3 protease. (**D**) Recombinant expression of recombinant plasmid pHT43-*htpX* in WB800N; 1: IPTG induction for 44 h; 2: without IPTG induction, 44 h. (**E**) Optimal temperature of the DX-3-*htpX* protease. (**F**) Optimal pH of the DX-3-*htpX* protease. The values represented the average of three independent experiments.

The strain *Priestia megaterium* DX-3, among those with protease activity extracted from the gut of *G. orientalis*, was fermented, and the supernatant was collected for electrophoresis analysis. The results showed that the 42 kDa protein band, i.e., the protein concentration increased by prolonging fermentation time (Figure 2A and Figure S2A), consistent with the dynamic trend in detected fermentation enzyme activity. The optimal reaction temperature of the DX-3 protease was 45 °C, which doubled the enzyme activity compared to 30 °C. The enzyme activity was lower at 60 °C and was greatly affected by temperatures above 60 °C. The DX-3 protease has a strong temperature tolerance, and the enzyme activity preservation rate was still over 90% at 50 °C for 8 h. However, it severely decreased at 60 °C for 8 h (Figure 2B). The optimal pH of the DX-3 protease was pH 7, and the enzyme activity was higher within the pH 7–9 range after storage in pH 6 buffer for 8 h. The enzyme activity preservation rate was the highest after storage in a pH 6 buffer for 8 h. However, the inactivation was more severe upon storage under acidic and alkaline conditions (Figure 2C). The DX-3 protease was neutral and heat-resistant.

The protease gene from strain DX-3 was recombined and expressed to explore the protease derived from gut bacteria. The primer was designed according to the protease gene *htpX* based on the whole genome sequencing of strain DX-3. We connected the *htpX* gene to the plasmid pHT43, then transformed it into *Bacillus subtilis* WB800N and constructed an engineered strain WB800N/pHT43-*htpX*. The recombinant strain was subjected to IPTG-induced fermentation in an LB medium and compared with the fermentation supernatant without IPTG induction for protein electrophoresis (Figure 2D and Figure S2B). The 42 kDa protein band was only expressed in the IPTG-induced process, indicating that the protease gene *htpX* was successfully expressed, and it was named DX-3-*htpX* protease.

After fermentation in LB medium, the DX-3-*htpX* protease exhibited a high fermentation activity of 135.68 ± 3.66 U/mL. In contrast, the fermentation activity of the DX-3 protease was relatively low, being about 2.19 ± 0.28 U/mL. The optimal reaction temperature for recombinant enzymes was still 45 °C, the enzyme activity increased by 250% from 30 °C to 45 °C (Figure 2E), and the optimal reaction pH was still 7. However, compared with the DX-3 protease, the high temperature resistance and the pH tolerance were improved (Figure 2F).

### 3.3. Homology Modeling and Binding Pocket Analysis of the DX-3-htpX Protease

**Figure 3:**
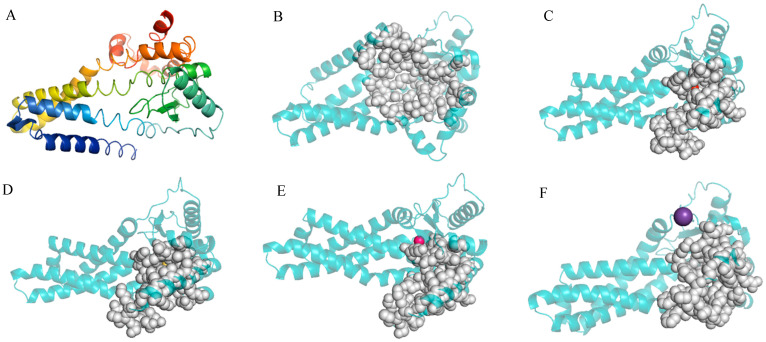
The three-dimensional structure and D3 pocket of the DX-3-*htpX* protease and its binding to different ions are shown by PyMOL. (**A**) The three-dimensional structure of the DX-3-*htpX* protease is shown as a cartoon and colored with a rainbow spectrum (N-terminal was purple, C-terminal was red). (**B**) The active site of the DX-3-*htpX* protease. (**C**) The active site of the DX-3-*htpX* protease binding to Ca^2+^. (**D**) The active site of the DX-3-*htpX* protease binding to Zn^2+^. (**E**) The active site of the DX-3-*htpX* protease binding to Cl^−^. (**F**) The active site of the DX-3-*htpX* protease binding to K^+^.

The entire length of the protease gene htpX coding sequence was 909 bp. It encoded a protein of 290 amino acid residues with no signal peptide. InterPro analysis of the htpX gene revealed that it contained 203 amino acid peptidase M48 domains (87–289) and metalloprotease (zincin) catalytic domains. The 3D structure of the DX-3-htpX protease was predicted using alphafold3, and the first-ranked model was selected as the research object according to the score ranking. PYMOL was used to display the three-dimensional junction of the DX-3-htpX protease (Figure 3A,B). The model of the DX-3-htpX protease consisted of ten α-helixes, four strands, two 310 helixes, twelve turns, seven bends, and multiple coil regions. The active site of the DX-3-htpX protease and its binding to different ions was predicted using CASTpFold (Figure 3C–F, Table 3), whereby it was shown that Ca^2+^, Zn^2+^, Cl^−^, and K^+^ binding to the DX-3-htpX protease could change the 3D structure and active sites of the DX-3-htpX protease. Ca^2+^, Zn^2+^, Cl^−^, and K^+^, in combination with the DX-3-htpX protease, could help to obtain a larger active pocket, among them, the binding of Ca^2+^ to the DX-3-htpX protease helped to obtain the largest active pocket.

## 4. Discussion

The primary method for exploring microbial enzymes is screening cultivable microorganisms [27]. Enzymes can be obtained after fermentation using screening culture media to enrich and isolate target microorganisms [28]. However, the above method has limitations with regard to studying unknown enzyme resources in non-cultivable microorganisms and complex samples. The insect gut is a complex ecosystem containing microorganisms, enzymes, and metabolites, and exploring gut microbial enzyme resources requires a series of non-cultivation techniques. The enzymes with proteolytic activity in mixed-protein samples from the gut of *G. orientalis* could be identified based on gelatin zymography [12]. Moreover, conventional protease inhibitors did not affect LGDBM-5 and LGDBM-6, indicating that these two protein samples may contain unannotated protease types. Although some information on gut proteases was obtained with the use of gelatin zymography and mass spectrometry technology, due to the sample size requirements of protein mass spectrometry technology, from the mass spectrometry process, some protease information relating to the low-concentration protein content in the insect gut may be missing [29], and further research is needed to develop a method for exploring gut enzyme resources that combines gelatin zymography and mass spectrometry.

Alternatively, we could mine enzyme genes based on genome sequencing and predict their catalytic mechanisms and functions [30]. Functional genes from microorganisms could also be cloned, and efficient biomass conversion systems could be used to prepare recombinant proteins [31]. In this study, protease genes derived from gut microbiota were recombined and expressed. The binding conformation of recombinant protease molecules with metal ions was simulated and compared, providing a data-anchored basis for optimizing the molecular conformation of recombinant protease to improve enzyme catalytic efficiency and facilitate the development and utilization of protease resources derived from the insect gut [8].

## 5. Conclusions

The protease resources derived from *G. orientalis* gut extract include cysteine proteases, pepsin, serine proteases, and trypsin, and *G. orientalis* gut extract may contain unrecognized proteases. Furthermore, the protease gene *htpX* derived from the gut bacteria DX-3 was cloned and recombinantly expressed. The DX-3-*htpX* protease was neutral, heat-resistant, and exhibited a metalloproteinase containing an M48 peptidase domain. The binding of Ca^2+^ to the DX-3-*htpX* protease could help to obtain the largest active pocket.

## Figures and Tables

**Table 1:** Prediction of protease components in gut protein samples based on protease inhibitors.

Gut Protease	Effective Protease Inhibitors	Protease Prediction
LGDBM-1	Pepsin inhibitor, PMSF, trypsin inhibitor	Pepsin, serine protease, trypsin
LGDBM-2	PMSF	Serine protease
LGDBM-3	Trypsin inhibitor	Trypsin
LGDBM-4	E-64, pepsin inhibitor, PMSF, trypsin inhibitor	Cysteine protease, pepsin, serine protease, trypsin
LGDBM-5	-	-
LGDBM-6	-	-

**Table 2:** Identification of protease types based on mass spectrometry.

Gut Protease	Gene Names	Protein Names	Accession No.	Protein Identification/%	Score
LGDBM-1		Glutathione S-transferase	P08515	35	141
	DCD	Dermcidin	P81605	19	110
	PSMA6	Proteasome subunit alpha type-6	P28072	7	74
LGDBM-3	DCD	Dermcidin	P81605	12	92
	FTSH8	ATP-dependent zinc metalloprotease	Q8W585	4	57
		Glutathione S-transferase	P08515	12	46
	GGCT	Gamma-glutamyl cyclotransferase	Q32LE4	5	33
LGDBM-4		Glutathione S-transferase	P08515	20	75
	DCD	Dermcidin	P81605	12	74
	groEL	Chaperonin GroEL	Q0KDR7	5	69
	CTSD	Cathepsin D	Q4LAL9	2	27
LGDBM-5	DCD	Dermcidin	P81605	12	74
		Glutathione S-transferase	P08515	26	66
	groEL	Chaperonin GroEL 1	A0K536	2	23
LGDBM-6		Glutathione S-transferase	P08515	26	110
	DCD	Dermcidin	P81605	12	85

**Table 3:** Active sites in the D3 pocket of the DX-3-*htpX* protease and their binding to different ions.

Protease	Area (Å^2^)	Volume (Å^3^)	Active Sites in the D3 Pocket
*HtpX*	557.472	837.241	ARG4, LEU7, PHE8, VAL11, ALA52, SER55, LEU56, SER59, MET62, ALA63, TRP65, MET66, MET67, ASN113, ALA114, PHE115, ALA116, THR117, GLY118, MET132, VAL144, HIS147, GLU148, HIS151, MET157, THR160, THR161, LEU162, GLN164, ILE214, HIS217, SER218, ARG221, GLU222, MET238, ALA241, LEU242, LEU254, THR276, HIS277, ARG283
*HtpX*-Ca^2+^	918.154	1378.221	LEU1, LYS3, ALA110, GLU111, VAL112, ASN113, ALA114, MET132, VAL144, HIS147, GLU148, ARG219, MET238, ALA241, LEU245, ARG246, THR248, THR249, SER250, VAL252, ASP253, GLN256, LYS257, ALA260, LYS263, ILE264, SER265, LYS267, GLU268, PHE270, SER271, ARG272, PHE274, SER275, HIS277, PRO278, PRO279, LEU280
*HtpX*-Cl^−^	714.286	867.364	LEU1, LYS3, GLU111, VAL112, ASN113, ALA114, MET132, VAL144, HIS147, GLU148, ARG219, MET238, ALA241, LEU242, LEU245, ARG246, THR248, THR249, SER250, VAL252, ASP253, GLN256, LYS257, ALA260, LYS263, ILE264, SER265, LYS267, GLU268, SER271, ARG272, SER275, HIS277, PRO278, PRO279, LEU280, GLU281, LYS282, ARG283
*HtpX*-K^+^	925.544	1335.237	LEU1, LEU2, LYS3, ALA110, GLU111, VAL112, ASN113, ALA114, MET132, VAL144, HIS147, GLU148, ARG219, MET238, ALA241, LEU242, LEU245, ARG246, THR248, THR249, SER250, VAL252, ASP253, GLN256, LYS257, ALA260, LYS263, ILE264, SER265, LYS267, GLU268, PHE270, SER271, ARG272, PHE274, SER275, HIS277, PRO278, PRO279, LEU280, GLU281, LYS282, ARG283
*HtpX*-Zn^2+^	811.023	1179.127	LEU1, LEU2, LYS3, ALA110, GLU111, VAL112, ASN113, ALA114, MET132, VAL144, HIS147, GLU148, ARG219, MET238, ALA241, LEU242, LEU245, ARG246, THR248, THR249, VAL252, ASP253, GLN256, LYS257, ALA260, LYS263, ILE264, SER265, LYS267, GLU268, SER271, ARG272, SER275, HIS277, PRO278, PRO279, LEU280, GLU281, ARG283

## Data Availability

Data generated during this study are available from the corresponding author upon reasonable request.

## Supplementary Materials

The following supporting information can be downloaded at: https://www.mdpi.com/article/10.3390/biology13090650/s1, Figure S1: The zymography analysis of the gut protein sample of *G. orientalis*. Figure S2: SDS-PAGE analysis of the DX-3 protease (A) and DX-3-htpX protease (B).
