# Supplementary material for: Exploration of Protease Resources in the Gut of Omnivorous Gryllotalpa orientalis (Orthoptera: Gryllotalpidae)
Source: Biology (Basel). 2024 Aug 23;13(9):650. doi: 10.3390/biology13090650 (PMC11428641; doi:10.3390/biology13090650)
Supplement: Supplementary file 1 [file biology-13-00650-s001.zip › biology-3119607-supplementary.pdf]

Supplemental Material

Exploration of protease resources in the gut of omnivorous *Gryllotalpa orientalis* (Orthoptera: Gryllotalpidae)

Xiang Zheng [1,2], Fangtong Wu [1], Lu Zhao [1], He Zhou [1], Zhijun Zhou [2], Zhenhua Jia [1,3] and Fuming Shi [2,*]

[1] Laboratory of Enzyme Preparation, Hebei Research Institute of Microbiology Co., Ltd, Baoding, 071051, China; 569186912@163.com; yayawu710@hotmail.com; 15832297069@139.com; ZhouHebio@163.com
[2] College of Life Science, Institute of Life Science and Green Development, Hebei University, Baoding, 071002, China; zhijunzhou@hbu.edu.cn; shif_m@126.com
[3] Institute of Biology, Hebei Academy of Sciences, Shijiazhuang, 050000, China; zhenhuaj@hotmail.com
- Correspondence: shif_m@126.com;

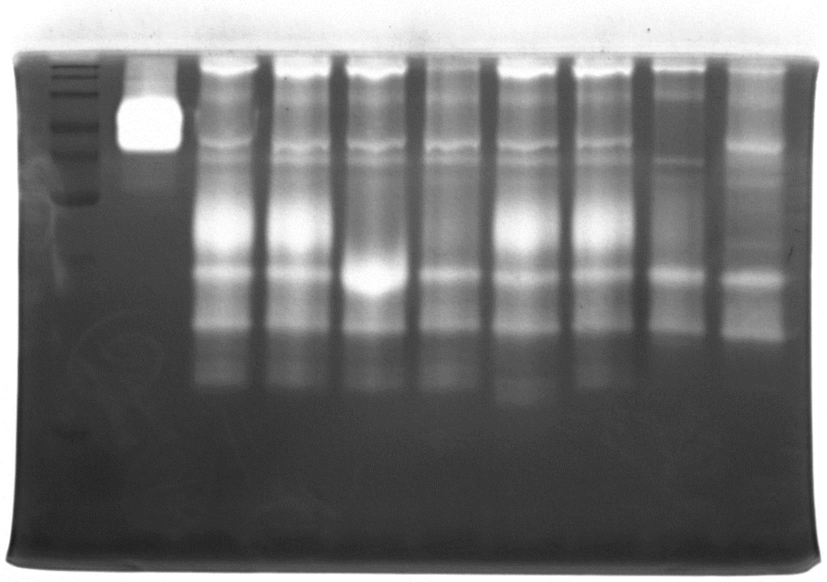

Supplemental Figure S1. The zymography analysis of the gut protein sample of *G. orientalis*.

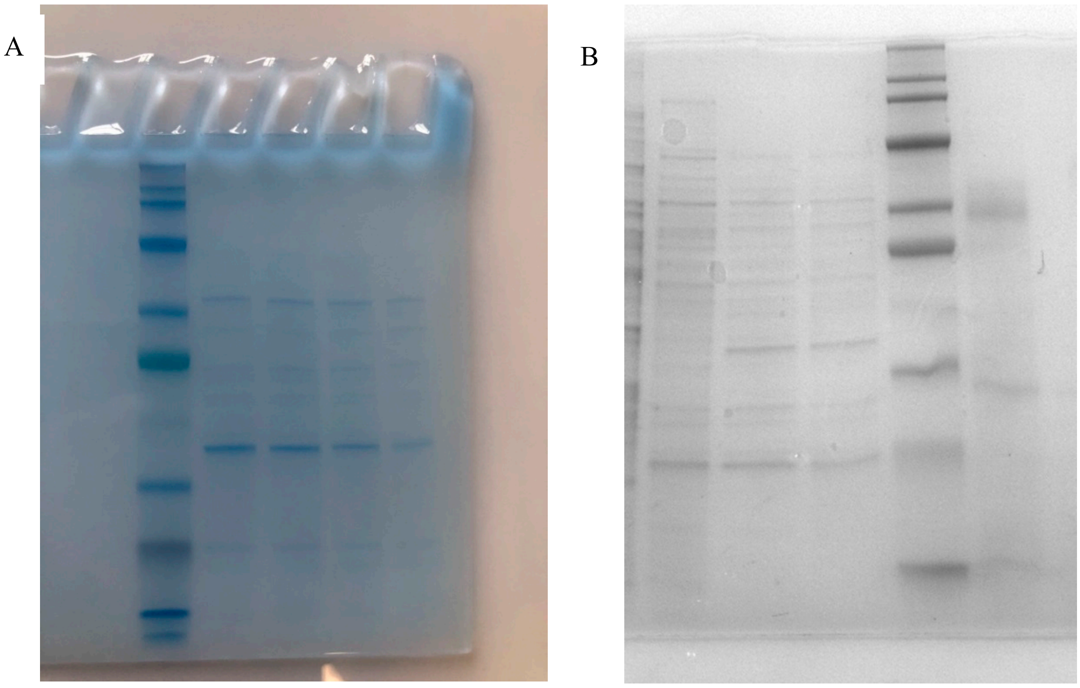

Supplemental Figure S2. SDS-PAGE analysis of DX-3 protease (A) and DX-3-*htpX* protease (B).
